# Dynamic variations in epithelial-to-mesenchymal transition (EMT), ATM, and SLFN11 govern response to PARP inhibitors and cisplatin in small cell lung cancer

**DOI:** 10.18632/oncotarget.15338

**Published:** 2017-02-15

**Authors:** C. Allison Stewart, Pan Tong, Robert J. Cardnell, Triparna Sen, Lerong Li, Carl M. Gay, Fatemah Masrorpour, You Fan, Rasha O. Bara, Ying Feng, Yuanbin Ru, Junya Fujimoto, Samrat T. Kundu, Leonard E. Post, Karen Yu, Yuqiao Shen, Bonnie S. Glisson, Ignacio Wistuba, John V. Heymach, Don L. Gibbons, Jing Wang, Lauren Averett Byers

**Affiliations:** ^1^ Department of Thoracic Head & Neck Medical Oncology, The University of Texas MD Anderson Cancer Center, Houston, TX 77030, USA; ^2^ Department of Bioinformatics and Computational Biology, The University of Texas MD Anderson Cancer Center, Houston, TX 77030, USA; ^3^ BioMarin Pharmaceutical, San Rafael, CA 94901, USA; ^4^ Department of Translational Molecular Pathology, The University of Texas MD Anderson Cancer Center, Houston, TX 77030, USA

**Keywords:** SCLC, SLFN11, ATM, EMT, PARP inhibitor

## Abstract

Small cell lung cancer (SCLC) is one of the most aggressive forms of cancer, with a 5-year survival <7%. A major barrier to progress is the absence of predictive biomarkers for chemotherapy and novel targeted agents such as PARP inhibitors. Using a high-throughput, integrated proteomic, transcriptomic, and genomic analysis of SCLC patient-derived xenografts (PDXs) and profiled cell lines, we identified biomarkers of drug sensitivity and determined their prevalence in patient tumors. In contrast to breast and ovarian cancer, PARP inhibitor response was not associated with mutations in homologous recombination (HR) genes (e.g., *BRCA1/2*) or HRD scores. Instead, we found several proteomic markers that predicted PDX response, including high levels of SLFN11 and E-cadherin and low ATM. SLFN11 and E-cadherin were also significantly associated with *in vitro* sensitivity to cisplatin and topoisomerase1/2 inhibitors (all commonly used in SCLC). Treatment with cisplatin or PARP inhibitors downregulated SLFN11 and E-cadherin, possibly explaining the rapid development of therapeutic resistance in SCLC. Supporting their functional role, silencing *SLFN11* reduced *in vitro* sensitivity and drug-induced DNA damage; whereas *ATM* knockdown or pharmacologic inhibition enhanced sensitivity. Notably, SCLC with mesenchymal phenotypes (i.e., loss of E-cadherin and high epithelial-to-mesenchymal transition (EMT) signature scores) displayed striking alterations in expression of miR200 family and key SCLC genes (e.g., *NEUROD1, ASCL1, ALDH1A1, MYCL1*). Thus, SLFN11, EMT, and ATM mediate therapeutic response in SCLC and warrant further clinical investigation as predictive biomarkers.

## INTRODUCTION

In patients with small cell lung cancer (SCLC), responses to frontline chemotherapy are rapidly overcome by drug resistance [1]. We previously demonstrated that PARP1 is a promising novel therapeutic target in SCLC [2, 3] and multiple PARP inhibitor clinical trials have now been initiated. However, unlike other forms of lung cancer, there are currently no biomarkers to predict treatment response in SCLC. In other cancers, response to PARP inhibitors has been associated with defects in DNA damage response (DDR) (e.g., mutations in *BRCA1/2, ATM*) [4, 5]. However, only ~12% of SCLC tumors have mutations in DDR genes [6–8] and most of those mutations do not have a known or predicted deleterious effect on function [6].

One emerging biomarker for PARP inhibitors and chemotherapy in several cancers is *Schlafen* 11 (SLFN11) [9–11]. We and others have recently described an association between SLFN11 expression and sensitivity to various PARP inhibitors, including talazoparib and olaparib, in SCLC models [12–14]. However, some models with low levels of *SLFN11* respond to PARP inhibition, whereas others with relatively high levels are resistant [12, 13]. These findings suggest that SLFN11 is unlikely to be the only determinant of drug response in SCLC.

To characterize SCLC-specific biomarkers of therapeutic vulnerability, we performed a high-throughput, integrated proteomic, transcriptomic, and genomic analysis using SCLC PDX models, cell lines, and archival tumor specimens. We found that while DDR mutations and HR deficiency (HRD) scores were not predictive in SCLC, expression levels of several markers including SLFN11, ATM, and E-cadherin (reflecting epithelial-to- mesenchymal transition (EMT) status) determined response to both PARP inhibitors and several classes of chemotherapy in preclinical models.

## RESULTS

### Identification of biomarkers in preclinical SCLC models

**Figure 1:**
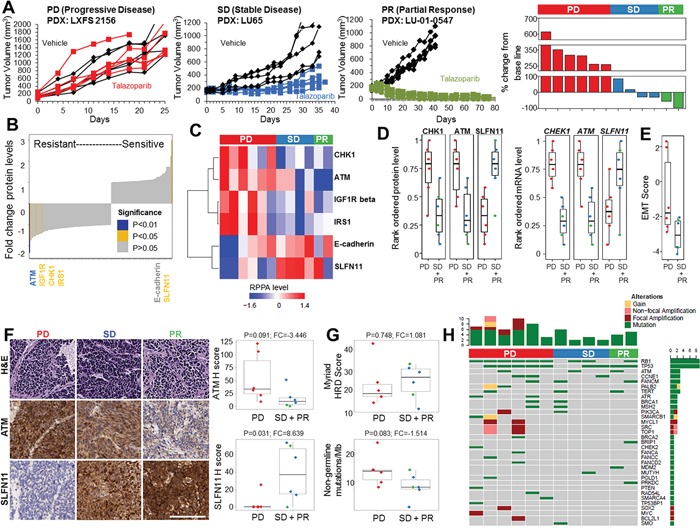
PDX Models with High SLFN11 and Low ATM Expression Levels Are More Sensitive to Talazoparib **A**. Examples of PDX response to single-agent talazoparib and percent change in tumor volume from baseline for individual PDX models. **B**, and **C**. Regression analysis of 12 PDX models grouped as having progressive disease (PD, n=6), stable disease (SD, n=4), or partial response (PR, n=2) following treatment with single-agent talazoparib identifies high SLFN11 and low ATM expression and CHK1, IGF1R beta, and IRS1 as markers of sensitivity. **D**. Analysis of CHK1, ATM, and SLFN11 protein and mRNA expression across response groups. **E**. EMT score is correlated with PDX resistance to talazoparib. **F**. Immunolocalization of SLFN11 and ATM in PDX models from the 3 response groups. SLFN11, but not ATM H score, predicts PDX response. **G**. Myriad HRD score and FoundationOne non-germline mutational burden do not predict PDX response to talazoparib. **H**. Oncoprint representation of FoundationOne mutations identified in DDR genes or mutations previously shown to occur in SCLC.

PDX models are derived from direct implantation of patient tumor biopsies into immunodeficient mice. PDX models retain both genetic similarities and drug response to the patient tumor from which they were developed and are useful for studying both tumor biology and preclinical testing [15, 16]. SCLC PDX models were treated with vehicle or talazoparib (PARP inhibitor) and assigned to response groups, partial response (PR), stable disease (SD), or progressive disease (PD) based on tumor growth and percent change from baseline (Figure 1A; Supplementary Figure 1). Tumors from untreated (vehicle) PDXs were analyzed by reverse-phase protein array (RPPA) and RNA sequencing (RNAseq) to identify pre-treatment protein and mRNA differences between those that were sensitive (PR or SD) or resistant (PD) to single-agent talazoparib. Of 170 proteins and/or phosphorylated proteins quantified by RPPA, low ATM (FC=−2.32, P=0.009) and high SLFN11 (FC=5.11, P=0.014) protein expression were the most significantly associated with talazoparib response in the PDX models (Figure 1B). High CHK1 (FC=−1.48, P=0.017), IGF1R beta (FC=−1.73, P=0.045), and IRS1 (FC=−1.39, P=0.025) protein levels were associated with resistance (Figure 1C). Protein biomarker results were further validated at the mRNA level which also showed an association between talazoparib response and high *SLFN11* (FC=38.82, P=0.031), low *ATM* (FC=−2.12, P=0.004), and low *CHEK1* (FC=−1.86, P=0.003) expression in PDX models (Figure 1D). Interestingly, although the two PDXs with the lowest ATM levels both had partial responses to talazoparib, only one of these expressed high levels of SLFN11 (Supplementary Figure 2C). This suggests that either low ATM or high SLFN11 is sufficient to predict sensitivity to talazoparib and therefore that more than one biomarker may predict SCLC response to PARP inhibition.

After SLFN11, E-cadherin (a marker commonly associated with EMT status) was the next strongest protein marker correlated with sensitivity (FC=2.24, P=0.158), although this did not reach statistical significance possibly due to the small sample size. Given previous findings by our group and others that EMT mediates resistance to several targeted therapies in non-small cell lung cancer (NSCLC), we further investigated the association between E-cadherin and talazoparib sensitivity using an EMT gene expression signature [17]. Consistent with the E-cadherin protein data, we found that PDX models that were resistant to talazoparib (PD) were more mesenchymal than those with PR or SD (mean difference=−2.21, P=0.052) (Figure 1E).

Finally, we performed immunohistochemistry (IHC) on tumors from the vehicle-treated PDXs for the top two biomarkers (SLFN11 and ATM) to independently validate the RPPA results (Figure 1F). H-scores for the ATM and SLFN11 levels were highly correlated with RPPA measurements (rho = 0.62 [P=0.02] and 0.82 [P=0.0006], respectively; Supplementary Figure 2D). As expected, SLFN11 H score was also correlated with PDX sensitivity (FC=8.639, P=0.03), whereas ATM H score was borderline significant (FC=−3.446, P=0.09; Figure 1F). This may have been due to the semi-quantitative nature of IHC compared with RPPA, which has greater sensitivity at lower protein expression levels [18]. These results suggest that IHC for SLFN11 and ATM in patient biopsy specimens could be a viable method for identifying patients with a higher likelihood of response to PARP inhibition.

### Biomarkers from other cancer types do not predict PARP inhibitor response in SCLC

In other cancers such as ovarian and breast, mutations in HR genes (e.g., *BRCA1/2, ATM*) and/or elevated HRD scores (e.g., Myriad Genetics testing scores) [19] predict patient response to PARP inhibitors. We found no association between PDX *in vivo* sensitivity to talazoparib and the HRD score (FC=1.081, P=0.748; Figure 1G). Consistent with this, HRD score also was not correlated with *in vitro* response to cisplatin (rho= −0.136, P=0.674), talazoparib (rho= 0.175, P=0.586) or olaparib (rho=0.290, P=0.360) (Supplementary Figure 2A) in a panel of 12 cell lines. We next tested whether the specific mutations were predictive in SCLC using a commercially available next-generation sequencing platform that includes targeted sequencing of 315 cancer-related genes plus select introns from 28 genes often rearranged or altered in solid tumor cancers (FoundationOne). PDX response did not correlate with the number of non-germline mutations (FC=−1.514, P=0.083; as determined by SGZ algorithm) (Figure 1G) or overall mutation burden (FC=−1.347, P=0.156; Supplementary Figure 2B). Furthermore, we found loss or mutation of *TP53* and/or *RB1* (hallmarks of SCLC) in the vast majority of PDX tumors; however, the mutations observed in genes previously associated with PARP inhibitor sensitivity were not predicted to be functionally relevant, nor were they significantly associated with talazoparib response *in vivo* (Figure 1H). These findings suggest that the ability of HR gene mutations and/or HRD scores to predict response to PARP inhibitors is disease-specific and unlikely to be a reliable method for selecting SCLC patients for treatment. Overall, we identified a number of new markers of sensitivity and resistance to PARP inhibitors in PDX models of SCLC (Table 1).

**Table 1:** Markers of talazoparib (PARP inhibitor) response in PDX models of SCLC

Markers of Talazoparib Response *in vivo* (PDX)			
	Marker	P-value	Fold Change
**Sensitivity**	SLFN11 (protein)	0.013	5.10
	*SLFN11* (mRNA)	0.031	38.82
	E-cadherin (protein)	0.158	2.24
	*CDH1* (mRNA)	0.137	7.11
**Resistance**	EMT score	0.05	−2.24(mean difference)
	ATM (protein)	0.009	−2.32
	*ATM* (mRNA)	0.004	−2.12
	CHK1 (protein)	0.017	−1.48
	*CHEK1* (mRNA)	0.003	−1.86
	IGF-1R (protein)	0.044	−1.73
	IRS1 (protein)	0.025	−1.39
**Not Associated**	Myriad HRD	0.748	1.081
	FMI Non-germline mutations	0.083	−1.514
	FMI All mutations	0.156	−1.347
	PAR (ELISA)	0.124	1.67
	PARP1 (protein)	0.614	−1.06

### SLFN11 and E-cadherin are the top biomarkers of PARP inhibitor response in SCLC cell lines

**Figure 2:**
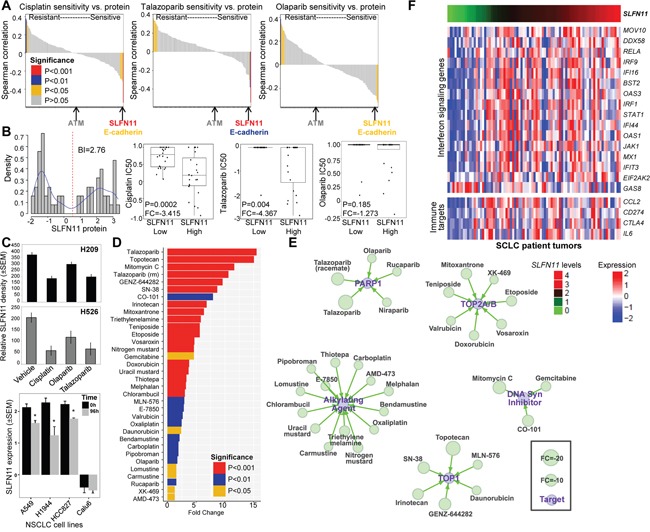
SLFN11 Protein Levels Are Correlated with Drug Sensitivity in SCLC **A**. A correlation analysis of cisplatin, talazoparib, and olaparib sensitivity (IC_50_ values) and 171 proteins in 51 SCLC cell lines shows that SLFN11 is the strongest predictor of sensitivity to both cisplatin and PARP inhibition. E-cadherin, but not ATM, is also correlated with drug sensitivity. **B**. SLFN11 expression is bimodal in SCLC cell lines, showing a switch-like pattern, and these naturally formed groups are correlated with drug sensitivity. Cell lines with higher SLFN11 protein expression levels have greater sensitivity to cisplatin and talazoparib but not olaparib. **C**. Treatment of H209 and H526 SCLC cell lines with 1 μM cisplatin, 1μM olaparib, or 100 nM talazoparib for 72h reduced SLFN11 levels compared with vehicle-treated cells*, P<0.0005. Treatment of A549, H1944, HCC827 NSCLC cell lines with 1 μM cisplatin for 96h reduced SLFN11 levels, but not Calu6. *, P<0.002. **D**. Waterfall plot of drug sensitivity in SCLC. A comparison of high SLFN11 protein levels and the IC_50_ values of 526 cancer drugs in 61 cell lines identified several drugs with similar targets, including PARP1 inhibitors, alkylating agents, TOP1 inhibitors, TOP2A/B inhibitors, and DNA synthesis inhibitors. **E**. A drug interaction network shows classes of oncology agents that are effective in SCLC cell lines with high SLFN11 levels. **F**. *SLFN11*-high SCLC has high expression levels of Type I IFN signaling molecules. IPA analysis identified IFN signaling as the top pathway associated with SLFN11 expression in SCLC patient tumors (P=6.6*10^−6^). Heatmap of Type I IFN signaling genes and immunotherapy target genes ranked by association with SLFN11 levels in 70 tumors from treatment-naïve SCLC patients (FDR=0.2).

Using an approach similar to the PDX analysis, we then used a panel of 51 SCLC cell lines to validate markers associated with drug sensitivity. Baseline expression of 170 proteins, including SLFN11 and ATM, were quantified by RPPA in untreated cell lines and correlated with sensitivity to PARP inhibitors (talazoparib, olaparib) and chemotherapy (cisplatin) (Figure 2A). As with the PDXs, for all 3 drugs, SLFN11 was the strongest marker of sensitivity by Spearman correlation (cisplatin, P<0.0001; talazoparib, P<0.0001; olaparib, P=0.02). In addition, E-cadherin was again identified as a top marker of sensitivity to cisplatin (P<0.007), talazoparib (P<0.002), and olaparib (P=0.021). Statistical analysis of the association between ATM levels and drug response was limited by the fact that only 4 cell lines in the panel had low ATM levels.

### SLFN11 predicts response to multiple drug classes and immune marker expression

SLFN11 protein and mRNA expression levels were highly concordant across the 51 SCLC cell lines (Rho=0.81, P<0.0001; Supplementary Figure 4A) and bimodally distributed (based on bimodal index [BI]=2.76 and 3.01, respectively; Figure 2B and Supplementary Figure 4A). Using the BI to define cell lines with high versus low SLFN11 levels [20], we found that cell lines with high SLFN11 protein expression were significantly more sensitive to cisplatin (FC=−3.415, P=0.0002) and talazoparib (FC=−4.367, P=0.004) but not olaparib (FC=−1.273, P=0.185; Figure 2B). Similarly, the high *SLFN11* mRNA expression group was also more sensitive to cisplatin (FC=−3.889, P=0.04), talazoparib (FC=−4.664, P=0.02), and olaparib (FC=−1.605, P=0.04).

Cell lines with high endogenous SLFN11 levels (H209 and H526) were treated with cisplatin, olaparib, or talazoparib to determine whether drug treatment alters SLFN11 expression. Treatment with cisplatin and both PARP inhibitors reduced SLFN11 (Figure 2C) and E-cadherin (Supplementary Figure 7D) levels as measured by relative density of western blots. Furthermore, cisplatin treatment of lung adenocarcinoma cell lines with high endogenous levels of SLFN11 showed a subsequent reduction in expression (A549, P=0.002; H1944, P<0.0001; HCC827, P=0.001), but not those with low levels (Calu6, P=0.785; Figure 2C). These data suggest SLFN11 levels in patient tumors may be similarly dynamic in response to treatment and, thus, biomarker analyses of initial diagnostic biopsies may be poor predictors of response to second or third line therapies.

Given the potential role of SLFN11 as a biomarker for several drug classes, we expanded our drug analyses of SCLC with high versus low SLFN11 expression levels to a larger screen of 526 oncology drugs in 63 SCLC cell lines [12]. We found that high SLFN11 protein levels, as determined by bimodal distribution, are associated with response to several drugs hitting the same targets or in common drug classes (Figure 2D and 2E). For example, high SLFN11 expression was correlated with sensitivity to alkylating agents (n=14), TOP1 inhibitors (n=6), TOP2A/B inhibitors (n=7), DNA synthesis inhibitors (n=3), and PARP inhibitors (n=4). Interestingly, although high SLFN11 expression was not correlated with olaparib response in our analysis (P=0.185), it was correlated with response to the drug in the analysis of the larger cell line panel (P=0.002). Although SLFN11 has been associated with sensitivity to drugs in these classes previously [9, 12, 13], ours is the most comprehensive list to date and includes additional drugs not previously reported. In contrast to SLFN11, none of the other *SLFN* family members (SLFN5, SLFN12, SLFN13, and SLFN14) were associated with *in vitro* response to cisplatin or talazoparib (Supplementary Figure 3A and Supplementary Figure 3B).

As with the cell lines, we also observed a bimodal distribution of SLFN11 in publically available mRNA data from 70 early-stage, treatment-naïve SCLC patient tumors (BI=1.46; Supplementary Figure 4A) [6], with 74% of tumors classified as *SLFN11*-high based on their BI. To better understand the functional role of SLFN11 in tumors, we performed Ingenuity Pathway Analysis (www.ingenuity.com) comparing genes associated with high vs. low *SLFN11* levels. Interestingly, these analyses revealed an enrichment of immune regulatory pathways, primarily interferon (IFN) signaling (P=6.6*10^−6^), in *SLFN11*-high tumors. Consistent with this, we found that *SLFN11* expression was positively correlated with Type I IFN pathway genes [21] in treatment-naïve SCLC patient tumors (Figure 2F). Furthermore, we screened a curated gene list enriched for immune targets [17] and found that high *SLFN11* expression was positively correlated with PDL1 (*CD274*; rho=0.248, P=0.025), *CCL2* (rho=0.271, P=0.014), *CTLA4* (rho=0.221, P=0.046), and *IL6* (rho=0.226, P=0.041) in these same patient tumors (Figure 2F). This association was not observed in SCLC cell lines and stimulation of SCLC cells with a Type I IFN *in vitro* did not strongly modulate SLFN11 expression (Supplementary Figure 6A), suggests that immune cell expression of SLFN11 in patient tumors may be important.

### Knockdown of SLFN11 and ATM directly alters drug sensitivity

**Figure 3:**
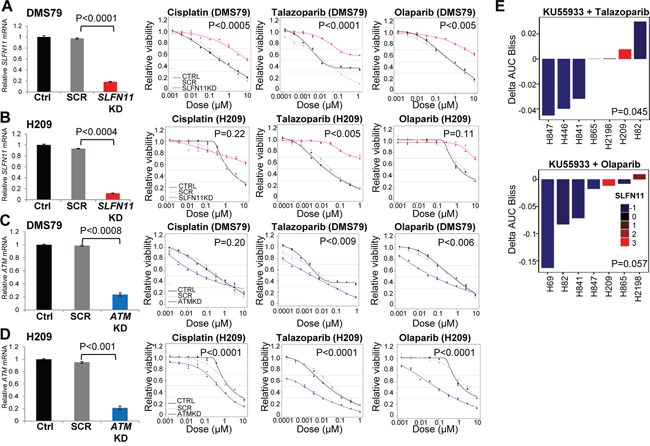
High SLFN11 and Low ATM Levels Maintain Sensitivity to PARP Inhibition and Chemotherapy **A**, and **B**. Silencing of *SLFN11* with siRNA in DMS79 and H209 SCLC cell lines (which have high SLFN11 expression levels, are sensitive to PARP inhibition, and have no *ATM* mutations), increases the cells’ resistance to cisplatin, talazoparib and olaparib. **C**, and **D**. *ATM* knockdown increases sensitivity to cisplatin, talazoparib, and olaparib. siRNA effectively reduced *SLFN11* and *ATM*. **E**. ATM inhibition sensitizes cell lines with low SLFN11 expression to PARP inhibition. The ATM inhibitor KU55933 plus talazoparib or olaparib was more effective in SCLC cell lines with lower SLFN11 levels.

Because of their roles in DNA repair [22, 23], we hypothesized that SLFN11 and ATM directly influence drug response in SCLC. H209 and DMS79 cell lines were selected for gene silencing based on their relatively high SLFN11 and ATM levels, and absence of *ATM* mutations. *ATM* mRNA and protein levels (Supplementary Figure 4C) were reduced by knockdown (P<0.0008 and <0.001, respectively; Figures 3C, 3D, and Supplementary Figure 4C), as were *SLFN11* mRNA and protein levels (P<0.0001 and <0.0004, respectively; Figures 3A, 3B, and Supplementary Figure 4C). Differences in expression of *ATM* and *SLFN11* were not present in control and scrambled samples for DMS79 or H209 (P>0.2). Silencing *SLFN11* increased the resistance of the DMS79 (Figure 3A) and H209 (Figure 3B) cell lines to cisplatin (P<0.0005, P=0.22), talazoparib (P<0.0001, P<0.005) and olaparib (P<0.005, P=0.119; Supplementary Table 1). Conversely, silencing of *ATM* increased the sensitivity of DMS79 (Figure 3C) and H209 (Figure 3D) to cisplatin (P=0.20, P<0.0001), talazoparib (P<0.009, P<0.0001) and olaparib (P<0.006, P<0.0001; Supplementary Table 1). These findings demonstrate that both SLFN11 and ATM levels play a direct role in regulating cisplatin and PARP inhibitor sensitivity/resistance in SCLC.

Based on our findings, we hypothesized that a small molecule inhibitor of ATM may sensitize SLFN11-low cell lines to PARP inhibition. Six SCLC cell lines (H82, H69, H841, H865, H2198, and H446) were treated with the ATM inhibitor KU55933 with or without the PARP inhibitors olaparib or talazoparib. The cell lines were not sensitive to treatment with the ATM inhibitor alone. However, in 5 of the 6 cell lines, the addition of the ATM inhibitor resulted in a modest sensitization to either of the PARP inhibitors. Furthermore, as predicted, the greatest sensitization was seen in cell lines with the lowest SLFN11 levels (P<0.05 for both PARP inhibitors; Figure 3E).

### High SLFN11 enhances DNA damage response

**Figure 4:**
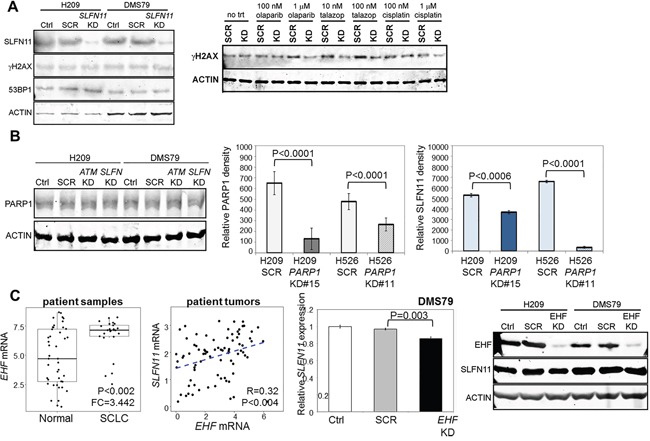
SLFN11 is Regulated by PARP1 and EHF **A**. Silencing of *SLFN11* in H209 and DMS79 is insufficient to induce the expression of H2AX, a marker of DNA damage. Stimulation with olaparib, talazoparib, and cisplatin induced γH2AX in scrambled (SCR) but not *SLFN11*-knockdown cells. **B**. Silencing of *SLFN11* or *ATM* does not affect PARP1 levels. Silencing of *PARP1* in H209 and H526 cells reduces SLFN11 levels. **C**. *EHF* expression is elevated in SCLC tumors compared to normal adjacent tissues and is correlated with *SLFN11* expression in tumors from treatment-naïve patients with early-stage SCLC. Silencing of *EHF* reduces *SLFN11* mRNA and protein expression levels.

We hypothesized that higher SLFN11 levels sensitize cells to DNA-damaging agents because of SLFN11′s roles in sequestering RPA and disrupting checkpoint maintenance and HR repair [22]. To test this hypothesis, we looked at markers of DDR after SLFN11 knockdown with and without low doses of PARP inhibitor or cisplatin treatment. Low doses were used to minimize cytotoxicity, so as to analyze DNA damage rather than induction of apoptosis. We found that *SLFN11* knockdown alone did not increase expression of γH2AX or change PARP1 levels (Figure 4A). However, the addition of treatment with PARP inhibitors or cisplatin for 24h prevented the induction of DNA damage (as indicated by γH2AX expression) (Figure 4A), suggesting that SLFN11 plays a direct role in cellular accumulation of DNA damage following stimulation.

### Function and regulation of SLFN11 in SCLC

Silencing of *SLFN11* or *ATM* did not alter PARP1 levels (Figure 4B), and PARP1 itself did not predict sensitivity to PARP inhibitors in our SCLC PDXs or cell lines. One mechanism of PARP inhibitor resistance that has been described for other cancer types is the downregulation of PARP1 itself [24], which we hypothesized directly affects SLFN11 levels. Thus, we generated cell lines with stable knockdown of *PARP1* with shRNA and assessed their SLFN11 levels. As shown by densitometric analysis of western blots, the knockdown cell lines had less SLFN11 (Figure 4B), regardless of the degree of PARP1 reduction. This suggests that decreased PARP1, as seen in some models of acquired resistance to PARP inhibitors, may play a role in regulating SLFN11 although further investigation is needed.

In other cancers, including Ewing sarcoma*, SLFN11* has been described as an ETS transcription factor response gene [25]. Thus, we tested whether similar regulation of SLFN11 occurs in SCLC. Expression of 26 ETS transcription factors was correlated with *SLFN11* and *PARP1* mRNA levels and with drug sensitivity (Supplementary Table 2). Of these, only *EHF* was correlated with *SLFN11* (P=0.048 in cell lines, p=0.004 in tumors; Supplementary Figure 6B) and with *in vitro* drug sensitivity (P=0.045; Supplementary Figure 6C). Like SLFN11, *EHF was* elevated in SCLC tumors (versus normal adjacent tissues) (Supplementary Table 3) and bimodally expressed (BI=2.66 in cell lines, 1.61 in tumors) [26]. Suppression of *EHF levels* reduced *SLFN11* expression (DMS79, P=0.003; Figure 4C). Overall, this suggests that the ETS transcription factors regulating SLFN11 may be cancer-specific.

SLFN11 levels are sensitive to hypermethylation of the *SLFN11* promoter [27]. To determine whether epigenetic methylation also plays a role in SLFN11 levels in SCLC, we analyzed 35 SCLC cell lines by Infinium HumanMethylation450 BeadChip array (Illumina). Levels of methylation in 19 CpG regions were linked to *SLFN11*. We identified 3 regions within the TSS that were negatively correlated with SLFN11 mRNA expression and 2 of these were also correlated with resistance to cisplatin, talazoparib, and olaparib (Supplementary Figure 8A-8D). The cg18608369 region, which has been reported to be correlated with cisplatin and carboplatin resistance in the NCI60 cell line panel [27], was not correlated with cisplatin resistance in SCLC (P=0.45). Treatment with a demethylase and/or HDAC inhibitor did not increase SLFN11 expression, and demethylation with decitabine did not improve SCLC cell lines’ sensitivity to cisplatin or PARP inhibitors (data not shown). Although SLFN11 promoter methylation is associated with cisplatin and PARP inhibitor resistance, demethylation and/or HDAC inhibition were not sufficient to upregulate SLFN11 levels (Supplementary Figure 8E). MGMT methylation was not strongly correlated with *SLFN11* expression or sensitivity to talazoparib or olaparib (data not shown).

### E-cadherin expression predicts drug sensitivity in SCLC

**Figure 5:**
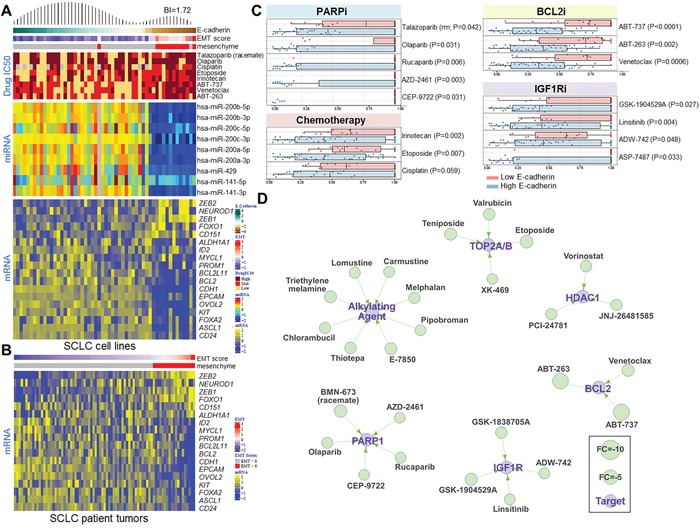
E-Cadherin Level Predicts Drug Sensitivity in SCLC **A**. Heatmap of SCLC cell lines ranked by E-cadherin protein levels demonstrating a bimodal distribution pattern (BI=1.72). Cell lines with EMT scores greater than 0 are classified as mesenchymal. Correlation of E-cadherin expression and the IC_50_ values of PARP inhibitors, standard-of-care chemotherapy drugs, and BCL2 inhibitors. miR200 family microRNAs are strongly correlated with E-cadherin levels and EMT score. Expression analysis of a subset of genes involved in SCLC progression that are correlated with E-cadherin expression. **B**. A heatmap of 70 tumor samples from treatment-naïve SCLC patients ranked by EMT score shows the expression analysis of the same subset of genes as in the SCLC cell lines. **C**. High E-cadherin levels are associated with sensitivity to several drug classes, including PARP inhibitors, chemotherapy drugs, BCL2 inhibitors, and IGF1R inhibitors. **D**. A drug interaction network shows classes of drugs that are effective in SCLC cell lines with high E-cadherin levels.

E-cadherin was a top marker of drug sensitivity in PDX models and cell lines (Figures 1B, 1C, and 2A). Therefore, to further establish whether the differences in E-cadherin reflected a broader EMT program, we investigated miRNAs and genes with established roles in EMT (e.g., miR200 family members, *ZEB1*, EMT gene signatures). Specimens were defined as mesenchymal if they had low E-cadherin protein (cell lines, as defined by their bi-modal index of 1.72) or a positive EMT gene signature score (patient tumors) (Figure 5A–5B) [6]. As expected, we observed a strong correlation between high E-cadherin levels and the miR-200 family members (r=0.29-0.83, corresponding to P<0.05-P<0.001), which are established repressors of EMT (Figure 5A) [28]. We then evaluated cell lines and SCLC patient tumors for association between high E-cadherin levels or low EMT scores, respectively and a curated list of genes known to be markers of cancer stem cells [29, 30], involved in EMT [31], and associated with tumor progression in SCLC [32]. In both cell lines and tumors, we found that high E-cadherin is inversely correlated with the EMT gene signature score (rho=−0.705, P<0.001 in cell lines, rho=−0.463, P<0.001 in tumors) (Figure 5A–5B). In general, epithelial cell lines and tumors were associated with cancer stem cell markers (*CD24*, *ALDH1A1*, and *PROM1*), markers of tumor aggressiveness *(MYCL1* and *ASCL1)*, and markers of apoptosis (*BCL2* and *BCL2L11*). However, mesenchymal cell lines and tumors were primarily associated with EMT markers (*ZEB1 and ZEB2)*. As SCLC is an extremely aggressive disease and *CDH1* levels are significantly higher in tumors than in normal adjacent tissues (FC=3.722; P<0.0001), the correlation between *CDH1* and these markers is not surprising (Supplementary Figure 7C).

Using an approach similar to that applied to SLFN11, we then investigated other drug classes for which E-cadherin might be a predictive marker of response in SCLC. Based on the bimodal index, we found that high E-cadherin level was associated with sensitivity to several drug types, including PARP inhibitors (n=5), alkylating agents (n=8), TOP2A/B inhibitors (n=4), HDAC1 inhibitors (n=3), BCL2 inhibitors (n=3), and IGF1R inhibitors (n=4) (Figure 5A–5D). Many of these drug classes overlap with those to which SLFN11-expressing SCLC cell lines are sensitive, although E-cadherin and SLFN11 levels were not strongly correlated (R=0.233, P=0.058; Supplementary Figure 7B), suggesting that EMT and SLFN11 contribute independently to drug response.

## DISCUSSION

Our findings suggest that high SLFN11 and low ATM expression levels predict PARP inhibitor response in PDX models and/or cell lines, whereas standard biomarkers of PARP inhibition defined in other cancer types (mutational burden, Myriad HRD score, and mutations in DDR genes) do not [33]. We also report for the first time that EMT score and E-cadherin levels predict response to PARP inhibition, chemotherapy, and other targeted drugs in development for SCLC. Thus, multiple biomarkers, including SLFN11, ATM, and E-cadherin, may be important in predicting PARP inhibition or chemotherapy response in SCLC.

Although low ATM levels were associated with talazoparib response in PDX models, we had an insufficient number of ATM-low cell lines to test its predictive value across our cell line panel. However, we found that *ATM* knockdown increased PARP inhibitor and cisplatin sensitivity, suggesting that loss of *ATM* expression has a direct impact on drug sensitivity. Further supporting this, we found that the combination of ATM and PARP inhibitors overcame resistance to single-agent PARP inhibition in lines with low SLFN11. These findings—together with recent clinical data showing that *ATM* mutations are associated with response to the PARP inhibitor olaparib in prostate cancer patients [34]—support the further investigation of ATM protein level as a candidate predictive biomarker. Moreover, inhibiting both ATM and PARP could help overcome drug resistance in patients whose tumors have low SLFN11 expression levels.

Based on levels of SLFN11 and ATM observed in treatment-naïve SCLC tumors, we expect that up to half of SCLC patients could respond to PARP inhibitors in the front-line setting. However, given that these cancers develop chemotherapy resistance within a few months after initial treatment, we predicted that SLFN11—a biomarker of cisplatin and PARP inhibitor response—may be downregulated following platinum-based chemotherapy. Indeed, cell lines treated with cisplatin or PARP inhibitors had reduced SLFN11 levels. This implies that archival (diagnostic) tumor tissue may not accurately predict response to PARP inhibitors in patients who have developed cisplatin resistance and, therefore, re-biopsy may be necessary to accurately assess biomarker status.

We found that SLFN11 directly regulated sensitivity to PARP inhibitors and cisplatin. Furthermore, loss of SLFN11 resulted in reduced DNA damage in response to cisplatin and PARP inhibitors, suggesting that its action is likely through regulation of DDR. Previous studies have shown that SLFN11 is recruited to sites of DNA damage, where it disrupts checkpoint maintenance and reduces HR repair [22]. We propose that high levels of SLFN11 may induce a type of synthetic lethality following PARP inhibitor or cisplatin treatment by negatively regulating DNA repair. Furthermore, based on our observation that silencing *PARP1* reduced SLFN11 levels, loss of PARP1 expression (as described in some models of acquired PARP inhibitor resistance [24]), may mediate the loss of drug sensitivity directly through SLFN11 downregulation.

SLFN11 levels directly confer chemotherapy and/or PARP inhibitor sensitivity in a number of cancer types, but it is unclear how this is controlled. Proposed mechanisms of SLFN11 regulation include IFN signaling [35], ETS transcription factor binding [25], and promoter methylation [27]. Several immune-related targets were found to be correlated with *SLFN11* in early-stage SCLC, including 16 Type I IFN signaling pathway genes and targetable immune markers *PDL1* and *CTLA4*. *SLFN11* is expressed by T-cells and monocytes and is an IFN-stimulated gene in peripheral blood mononuclear cells [36]. However, SLFN11 is not IFN-stimulated in SCLC cell lines suggesting that expression and regulation of SLFN11 by tumor and immune cells may be different. *SLFN11* is also a potential surrogate marker of T-cell or macrophage infiltration and a biomarker for response to immunotherapy drugs targeting PDL1 and CTLA4, but this requires further testing in a clinical setting. Given the recent addition of immunotherapy to treatment options for relapsed SCLC (www.NCCN.org), these findings merit further investigation given their potential clinical impact [37]. The *SLFN11* promoter contains several ETS binding sites and functions as an ETS factor response gene, with ETS transcription factors *FLI1* and *ETS1* having proposed roles in regulating *SLFN11* expression in colon, breast, and prostate cancers [25]. In our analysis of ETS family members, only *EHF* is elevated in SCLC tumors, positively correlated with SLFN11 response (cell line and tumors) and predicts drug response. In contrast, *EHF* is negatively correlated with *SLFN11* expression in other cancers from NCI60 and CCLE data sets [25]. These findings imply that although *SLFN11* functions as an ETS response gene, the particular ETS transcription factor that regulates *SLFN11* expression is cancer type–specific. In agreement with previous studies showing that hypermethylation regulates SLFN11 and is correlated with cisplatin sensitivity in NCI60 cell lines [27], we found two CpG regions near the SLFN11 TSS to be inversely correlated with SLFN11 mRNA levels and with *in vitro* sensitivity to both cisplatin and PARP inhibitors. However, treatment with decitabine and/or an HDAC inhibitor was insufficient to raise SLFN11 levels in SCLC cell lines. This suggests that methylation or other epigenetic modifications may not be dominant regulators of SLFN11 levels in SCLC, but may be useful as a biomarker of response to both cisplatin and PARP inhibitors.

We and others have previously shown an important role for EMT in modulating drug resistance in NSCLC. However, this is the first report linking EMT with resistance to multiple drug classes in SCLC. We found a significant association between EMT and resistance to PARP inhibitors, cisplatin, and other novel drug classes such as Bcl-2 inhibitors. In SCLC lines and patient tumors, loss of E-cadherin and high EMT gene signature score were associated with changes at the protein, miRNA, and mRNA level in genes and miRNA well known to regulate EMT, further supporting the presence of a mesenchymal subset of SCLC. This included downregulation of miR-200 family members (important regulators of EMT, metastasis, and disease recurrence in NSCLC) [28, 38]; higher *ZEB1/2, FOXO1*, and *NEUROD1*; and decreased levels of stem cell markers (e.g., *ALDH1A1*) and other SCLC drivers (e.g., *MYCL1*, *ASCL1)* [32, 39]. ASCL1 and NEUROD1 are both necessary for SCLC tumor survival and disease progression, but regulate different downstream pathways, and whether they are involved in EMT is unknown [32].

To further explore the association between EMT and drug resistance, we expanded our analysis to a large screen of oncology drugs [12]. In addition to PARP inhibitors, this analysis revealed greater resistance of mesenchymal SCLC to alkylating agents, TOP2A/B inhibitors, BCL2 inhibitors, IGF1R inhibitors, and HDAC1 inhibitors. The higher response rate of E-cadherin high SCLC to BCL2 inhibitors (e.g., ABT-737, ABT-263, venetoclax) corresponded with higher BCL2 mRNA expression in the E-cadherin high cell lines and tumors, suggesting that BCL2 levels or EMT status could be used to help select patients for treatment with BCL-2 inhibitors and that pharmacologic strategies to reverse EMT may enhance BCL-2 inhibitor activity.

In conclusion, our findings support the notion that more than one biomarker is needed to predict SCLC response to PARP inhibition and/or chemotherapy and that expression of these biomarkers is dynamically regulated by drugs used in frontline therapy. In addition, our results show that SLFN11 and ATM directly regulate drug sensitivity, which suggests that therapeutically targeting these proteins may reduce or delay drug resistance in SCLC. Our results also show that the dependence of SLFN11 levels on *PARP1* may represent a novel mechanism of acquired resistance to PARP inhibitors. Finally, E-cadherin levels and EMT score are linked with SCLC response to PARP inhibitors, chemotherapy, and other targeted therapies. Future studies should investigate methods to restore SLFN11 levels in resistant models and mechanisms regulating EMT, in order to develop means to therapeutically alter EMT while inhibiting PARP in SCLC.

## MATERIALS AND METHODS

### Histology and IHC of PDX models

A pathologist specializing in lung cancer (J.F.) reviewed each PDX model for hematoxylin and eosin and standard SCLC IHC markers (CD56, chromogranin A, and synaptophysin) to confirm that tumor pathology was consistent with SCLC.

IHC markers assessed in this study included those used routinely in the clinical diagnosis and characterization of lung cancers (synaptophysin, CD56, chromogranin A, Ki-67, and TTF1) and candidate biomarkers (SLFN11, ATM, and PARP).

### Mutation analysis

DNA from PDX tumors was profiled using the clinical comprehensive genomic profiling FoundationOne hybrid-capture based NGS test (Foundation Medicine, Inc., Cambridge, MA) [40]. Mutation load was extrapolated to the exome or genome and estimated. The mutation load per megabase was calculated by dividing the total number of counted mutations by the coding region of the test (1.110 megabases). Foundation Medicine's SGZ algorithm was used to predict germline mutations [41]. Genomic instability was predicted for the PDX tumors and for 12 SCLC cell lines using the myChoice HRD assay (Myriad Genetics, Inc., Salt Lake City, UT).

### Cell lines

Cells were grown in suggested media supplemented with fetal bovine serum and penicillin/streptomycin. Cells were passaged less than 6 months from the time they were received, and cell lines were routinely subjected to DNA fingerprinting as described previously [2].

### Gene knockdown

For gene silencing, pooled small interfering RNAs (siRNAs) targeting *SLFN11* (L-016764–01-0005) or *ATM* (L-003201-00-0005) and their corresponding scramble control (D-001810–10-05; GE Dharmacon) or *EHF* (S25399) and the Silencer Select Negative Control No. 2 (4390846, ThermoFisher Scientific) were transfected into H209 and DMS79 cells for 72 hours. The cells were then plated for 7-day proliferation assays or 24-h single-agent drug treatment. Knock down efficiency was validated by quantitative reverse transcription polymerase chain reaction and western blot analyses. *PARP1* was stably knocked down in H209 and H526 cell lines using plasmid vectors. The *PARP1* shRNAs were from GE Dharmacon (V3LHS_391011, V3LHS_391013). Knock down efficiencies were assessed with western blotting.

### Drug treatments

To determine effect of drug treatment on protein expression, H209 and H526 cells were plated for 24 h and then treated with 1 μM cisplatin, 1 μM olaparib, 100 nM talazoparib. Cells were collected 72 h later, and protein lysates were isolated. SLFN11, E-cadherin, and ATM expression were assessed by western blotting. For the demethylation/HDAC inhibition experiments, H69, H526, H841, and H209 cells were plated for 24 h and then treated for 72 h with dimethyl sulfoxide or 5 μM decitabine (Sigma Aldrich, St. Louis, MO) or with 100 ng/ml trichostatin A (Sigma Aldrich) added in the last 16h. All experiments were performed in triplicate.

## SUPPLEMENTARY MATERIALS FIGURES AND TABLES


