# Supplementary material for: Dynamic variations in epithelial-to-mesenchymal transition (EMT), ATM, and SLFN11 govern response to PARP inhibitors and cisplatin in small cell lung cancer
Source: Oncotarget. 2017 Feb 15;8(17):28575–87. doi: 10.18632/oncotarget.15338 (PMC5438673; doi:10.18632/oncotarget.15338)
Supplement: Supplementary file 1 [file oncotarget-08-28575-s001.pdf]

Dynamic variations in epithelial-to-mesenchymal transition (EMT), ATM, and SLFN11 govern response to PARP inhibitors and cisplatin in small cell lung cancer

SUPPLEMENTARY DATA

PDX model

Twelve PDX models of SCLC obtained from Crown Biosciences, OncoTest, WuxiAppTec, or Champions Oncology were evaluated for their response to single-agent talazoparib. PDX tumors were propagated subcutaneously in immunocompromised BALB/c nude, NOD/SCID, or NMRI nude female mice at passage 3 to 13. Due to the use of different genetic backgrounds for PDX models, it is possible that there may be potential variations in pharmacokinetics and tumor microenvironment. When tumors reached an average volume of about 150 mm^3, animals (n=5 per group) were orally administered vehicle with or without the maximum tolerated dose of talazoparib (0.25–0.3 mg/kg) daily. Tumor volume and animal body weight were measured twice weekly until the end of study or when tumor size reached 2000 mm^3. The median tumor volume on day 21 and beyond first treatment was used to calculate the change from baseline to identify best response. PDX tumors (~150 mm^3) collected from untreated tumor-bearing mice were used for histology and profiling experiments. Based on tumor size change from baseline, PDXs were classified as having a partial response (<30% change; n=2), stable disease (SD; 30%–100%; n=4), or progressive disease (PD; >100%; n=6). Poly-ADP-Ribose (PAR) enzyme-linked immunosorbent assay was performed as described previously [1] to measure PARP1 activity.

Immunohistochemistry

The diagnosis of small cell lung cancer (SCLC) can be determined by hematoxylin and eosin stain in most cases, but the most useful neuroendocrine markers are CD56, chromogranin, and synaptophysin [2]. Four-micrometer-thick tissue sections were cut for immunohistochemistry (IHC). IHC staining was performed with a Bond Max automated staining system (Leica Microsystems Inc., Vista, CA) using IHC parameters optimized previously. Leica Bond Retrieval Solution #2 (pH9) was used in SLFN11, ATM, and PARP staining studies, and Bond Retrieval Solution #1 (pH6) was used in other staining studies. The antibodies used in this study included antibodies against SLFN11 (Sigma-Aldrich, St. Louis, MO; 1:50 dilution), ATM (clone #Y170, Abcam, Cambridge, UK, 1:300 dilution), PARP (Thermo Scientific, Waltham, MA, 1:100 dilution), Ki67 (clone #D2H10, Cell Signaling Technology, Danvers, MA, 1:400 dilution), TTF1 (clone #EPR5955 [2], Abcam, 1:400 dilution), synaptophysin (clone #SP11, Spring Bioscience, Pleasanton, CA, 1:100 dilution), CD56 (clone #EP2567Y, Abcam, 1:50 dilution), and chromogranin A (clone #LK2H10, EMD Millipore [Chemicon], Billerica, MA, 1:4000).

Staining was developed with chromogen substrate (Leica Microsystems Inc.) and then counterstained with hematoxylin, dehydrated, and mounted. The stained slides were digitally scanned using the Aperio ScanScope Turbo slide scanner (Leica Microsystems Inc.). The digital images were captured at ×200 magnification. The images were visualized using ImageScope software (Leica Microsystems, Inc.), and digital image analysis was performed using the Aperio Image Toolbox (Leica Microsystems Inc.). Nuclear expression of SLFN11 and ATM were quantified using a 4-value intensity score (0, none; 1, weak; 2, moderate; and 3, strong) and the percentage (0%–100%) of the extent of reactivity. A final expression score (H-score) was obtained by multiplying the intensity and reactivity extension values (range, 0–300) as described previously [3, 4]. All IHC markers were validated and optimized using positive and negative controls. The specimens were stained using the same IHC conditions with a Bond Max automated staining system (Leica Microsystems, Inc.) simultaneously with positive and negative controls. IHC data were examined by 2 experienced pathologists (JF and IW).

Cell lines

The human SCLC cell lines H446, CORL88, DMS 114, DMS 153, DMS 273, DMS 53, DMS 79, H1048, H1092, H1105, H1184, H1238, H128, H1341, H1417, H1436, H146, H1522, H1618, H1672, H1688, H1694, H1836, H187, H1876, H1930, H196, H1963, H2029, H2081, H209, H2107, H2108, H211, H2141, H2171, H2195, H2196, H2198, H220, H2227, H2330, H250, H345, H378, H510A, H524, H526, H660, H69, H69/CR, H719, H735, H740, H774, H748, H82, H841, H847, H865, H889, and SHP-77 were obtained from ATCC (Manassas, VA), from Sigma Aldrich, or from Drs. John Minna and Adi Gazdar (UT Southwestern, Dallas, TX). The patient-derived xenograft cell line NJH29 was kindly provided by Dr. Julien Sage (Stanford University, Stanford, CA).

RPPA

Protein lysates from tumors and cell lines were quantified and protein arrays were printed and stained as described previously [3]. Images were quantified with MicroVigene 4.0 (VigeneTech, Carlisle, MA). The spot-level raw data were processed with the R package SuperCurve suite, which returns the estimated protein concentration (raw concentration) and a quality control score for each slide, as described previously [3]. Only slides with a quality control score of >0.8 were used for downstream analysis. The raw concentration data were normalized by median-centering each sample across all the proteins to correct loading bias.

Proliferation assays

Cells were seeded in 96-well plates at 2,000 cells per well. After 24 h, the cells in each well were treated with dilutions of cisplatin, talazoparib, olaparib, KU55993, or vehicle control. In the drug combination experiments, the cells received equimolar doses of both drugs; the doses were the same as those used for the single-agent treatments. Proliferation of the single- and combination-treatment SCLC cell lines were assayed by Cell Titer Glo (Promega, Fitchburg, WI) after 96 hours. IC_{50} values were estimated with drexplorer software [5].

RNA sequencing analysis of patient-derived xenografts

The reference genome and gene annotation for human (GRCh38, version 20) [6] and mouse (GRCm38, version M4) [7] were downloaded from GENCODE (http://www.gencodegenes.org/). RNA sequencing reads for patient-derived xenografts (PDX) tumors were aligned to the combined human and mouse genome with STAR version 2.4.1b [8] with 2-pass mapping and alignments with non-canonical junctions filtered out. The resulting alignment BAM files were sorted by read using SAMtools version 1.2 [9]. HTSeq version 0.6.1p1 [10] was used to count reads for each gene in the combined human and mouse gene annotation. Only reads that could be uniquely aligned to a human gene were counted to represent the gene's expression.

microRNA arrays

Total RNA from 51 cell lines was analyzed with Affymetrix miRNA 4.0 arrays. The expression data of a curated list of miR-200 family members (hsa-miR-200b-5p, hsa-miR-200b-3p, hsa-miR-200c-5p, hsa-miR-200c-3p, hsa-miR-200a-5p, hsa-miR-200a-3p, hsa-miR-429, hsa-miR-141-5p, hsa-miR-141-3p) known to be involved with epithelial–mesenchymal transition (EMT) in NSCLC [11] were compared with E-cadherin expression data.

Methylation microarrays

To determine the methylation status of SLFN11 and ATM, we compared 31 SCLC cell lines using the Infinium HumanMethylation450 BeadChip array (Illumina, San Diego, CA) with 485,577 probes. The methylation level of each genomic region is represented by a β-value ($0 \leq \beta \leq 1$), where 0 represents unmethylated and 1 represents fully methylated. These methylation values were compared with mRNA expression data and the median inhibitory concentration (IC_{50}) values for cisplatin, talazoparib, and olaparib.

Calculation of drug parameters

For single-agent analysis, the IC_{50} value was estimated using the drexplorer software program, which fitted multiple dose-response models and selected the best model using residual standard error [5]. For drug combination analysis, the Bliss independence model was used to estimate the additive effect [12]. Area under curve (AUC) was calculated for the curve from additive effect (AUC_0) and drug combination (AUC_1). ΔAUC was defined as the difference between AUC_1 and AUC_0 ($\Delta AUC = AUC_1 - AUC_0$). Therefore, a negative ΔAUC value suggests a more-than-additive effect, and a positive ΔAUC value suggests a less-than-additive effect.

Statistical analysis

For purposes of our exploratory protein analyses, a p value <0.05 was used to select candidate protein biomarkers. Spearman rank correlation was used to assess the association between 2 continuous variables, which included IC_{50} values, protein expression (as measured with reverse-phase protein array analysis), mRNA expression, and various scores. The Student t-test was used to assess the association between a binary variable (such as response group and tissue type) and a continuous variable. Bimodal expression was assessed using the Bimodality Index, an algorithm developed in house [13, 14]. EMT score was calculated based on the EMT signature as described previously [15]. The drug-target interaction graph was constructed with the Fruchterman-Reingold algorithm [16] implemented in the iGraph R package [17]. P values were calculated using 2-sided tests. The candidate protein markers were selected by top-ranked proteins with P values of less than 0.05. All statistical analyses were performed using the R software program [18].

REFERENCES

1. Cardnell RJ, Feng Y, Diao L, Fan YH, Masrorpour F, Wang J, Shen Y, Mills GB, Minna JD, Heymach JV, Byers LA. Proteomic markers of DNA repair and PI3K pathway activation predict response to the PARP inhibitor BMN 673 in small cell lung cancer. Clin Cancer Res. 2013; 19: 6322-8. doi: 10.1158/1078-0432.CCR-13-1975.
2. Travis WD. Update on small cell carcinoma and its differentiation from squamous cell carcinoma and other non-small cell carcinomas. Mod Pathol. 2012; 25 Suppl 1: S18-30. doi: 10.1038/modpathol.2011.150.
3. Byers LA, Wang J, Nilsson MB, Fujimoto J, Saintigny P, Yordy J, Giri U, Peyton M, Fan YH, Diao L, Masrorpour F, Shen L, Liu W, et al. Proteomic profiling identifies dysregulated pathways in small cell lung cancer and novel therapeutic targets including PARP1. Cancer Discov. 2012; 2: 798-811. doi: 10.1158/2159-8290.CD-12-0112.
4. Fujimoto J, Kadara H, Garcia MM, Kabbout M, Behrens C, Liu DD, Lee JJ, Solis LM, Kim ES, Kalhor N, Moran C, Sharafkhaneh A, Lotan R, et al. G-protein coupled receptor family C, group 5, member A (GPRC5A) expression is decreased in the adjacent field and normal bronchial epithelia of patients with chronic obstructive pulmonary disease and non-small-cell lung cancer. J Thorac Oncol. 2012; 7: 1747-54. doi: 10.1097/JTO.0b013e31826bb1ff.
5. Tong P, Coombes KR, Johnson FM, Byers LA, Diao L, Liu DD, Lee JJ, Heymach JV, Wang J. drexplorer: A tool to explore dose-response relationships and drug-drug interactions. Bioinformatics. 2015; 31: 1692-4. doi: 10.1093/bioinformatics/btv028.
6. Harrow J, Frankish A, Gonzalez JM, Tapanari E, Diekhans M, Kokocinski F, Aken BL, Barrell D, Zadissa A, Searle S, Barnes I, Bignell A, Boychenko V, et al. GENCODE: the reference human genome annotation for The ENCODE Project. Genome Res. 2012; 22: 1760-74. doi: 10.1101/gr.135350.111.
7. Mudge JM, Harrow J. Creating reference gene annotation for the mouse C57BL6/J genome assembly. Mamm Genome. 2015; 26: 366-78. doi: 10.1007/s00335-015-9583-x.
8. Dobin A, Davis CA, Schlesinger F, Drenkow J, Zaleski C, Jha S, Batut P, Chaisson M, Gingeras TR. STAR: ultrafast universal RNA-seq aligner. Bioinformatics. 2013; 29: 15-21. doi: 10.1093/bioinformatics/bts635.
9. Li H, Handsaker B, Wysoker A, Fennell T, Ruan J, Homer N, Marth G, Abecasis G, Durbin R, Genome Project Data Processing S. The Sequence Alignment/Map format and SAMtools. Bioinformatics. 2009; 25: 2078-9. doi: 10.1093/bioinformatics/btp352.
10. Anders S, Pyl PT, Huber W. HTSeq--a Python framework to work with high-throughput sequencing data. Bioinformatics. 2015; 31: 166-9. doi: 10.1093/bioinformatics/btu638.
11. Chen L, Gibbons DL, Goswami S, Cortez MA, Ahn YH, Byers LA, Zhang X, Yi X, Dwyer D, Lin W, Diao L, Wang J, Roybal JD, et al. Metastasis is regulated via microRNA-200/ZEB1 axis control of tumour cell PD-L1 expression and intratumoral immunosuppression. Nat Commun. 2014; 5: 5241. doi: 10.1038/ncomms6241.
12. Bliss CI. The toxicity of poisons applied jointly. Annals of Applied Biology. 1939; 26: 585-615. doi: 10.1111/j.1744-7348.1939.tb06990.x.
13. Tong P, Chen Y, Su X, Coombes KR. SIBER: systematic identification of bimodally expressed genes using RNAseq data. Bioinformatics. 2013; 29: 605-13. doi: 10.1093/bioinformatics/bts713.
14. Wang J, Wen S, Symmans WF, Pusztai L, Coombes KR. The bimodality index: a criterion for discovering and ranking bimodal signatures from cancer gene expression profiling data. Cancer Inform. 2009; 7: 199-216. doi:
15. Byers LA, Diao L, Wang J, Saintigny P, Girard L, Peyton M, Shen L, Fan Y, Giri U, Tumula PK, Nilsson MB, Gudikote J, Tran H, et al. An epithelial-mesenchymal transition gene signature predicts resistance to EGFR and PI3K inhibitors and identifies Axl as a therapeutic target for overcoming EGFR inhibitor resistance. Clin Cancer Res. 2013; 19: 279-90. doi: 10.1158/1078-0432.CCR-12-1558.
16. Fruchterman TMJ, Edward, M.R. Graph drawing by force-directed placement. Software Practice and Experience. 1991; 21: 1991. doi: 10.1002/spe.4380211102.
17. Csardi G NT. The igraph softward package for complex network research. InterJournal. 2006; Complex Systems: 1695. doi:
18. Team RC. (2015). R: A language and Environment for Statistical Computing. In: Computing RFfS, ed. (Vienna, Austria.

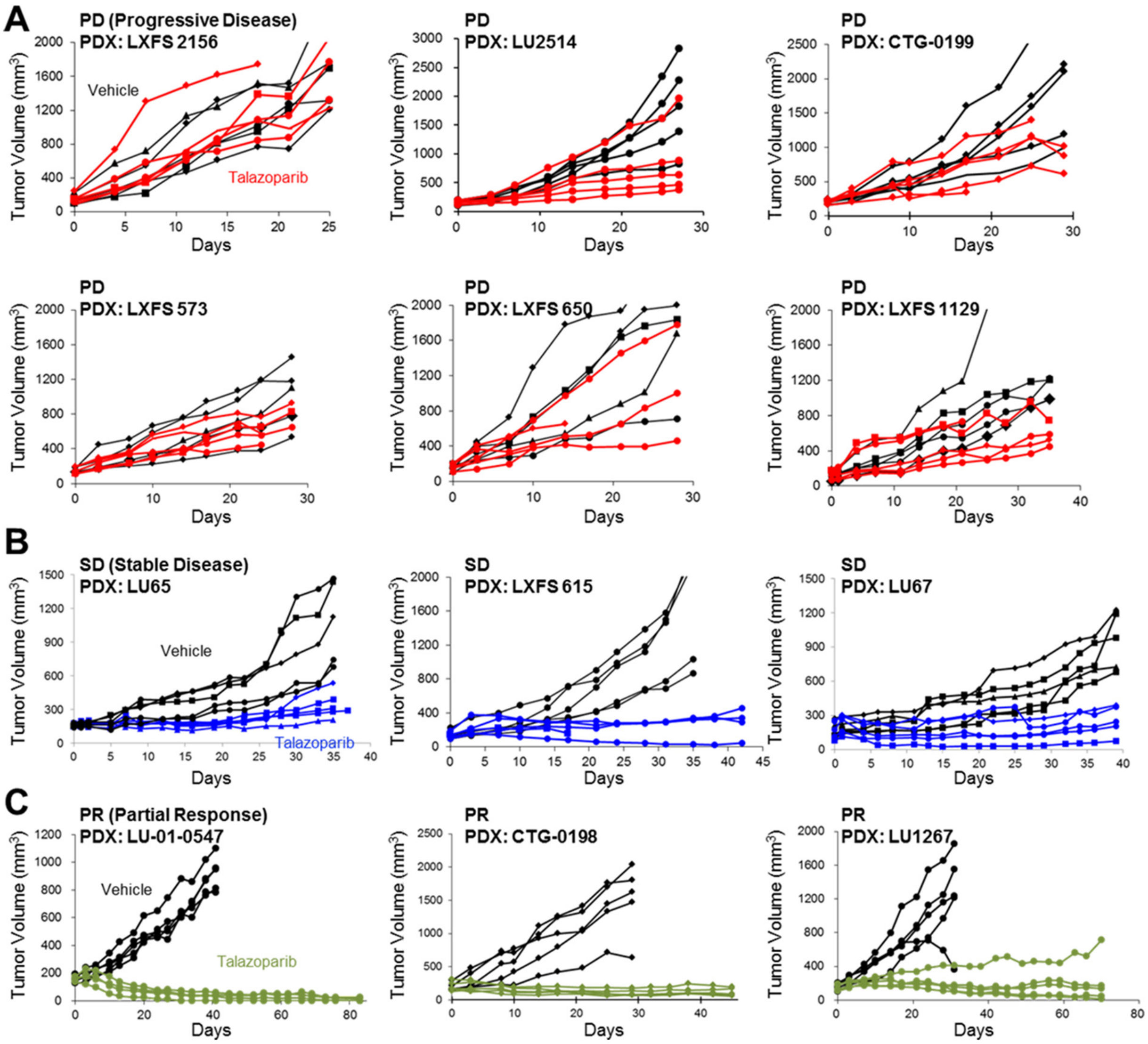

Supplementary Figure 1: Tumor growth curves for all PDX tumors treated with talazoparib. A. Tumor volume measurements for PDX models classified as Progressive Disease (PD). **B.** Tumor volume measurements for PDX models classified as Stable Disease (SD). **C.** Tumor volume measurements for PDX models classified as Partial Responders (PR).

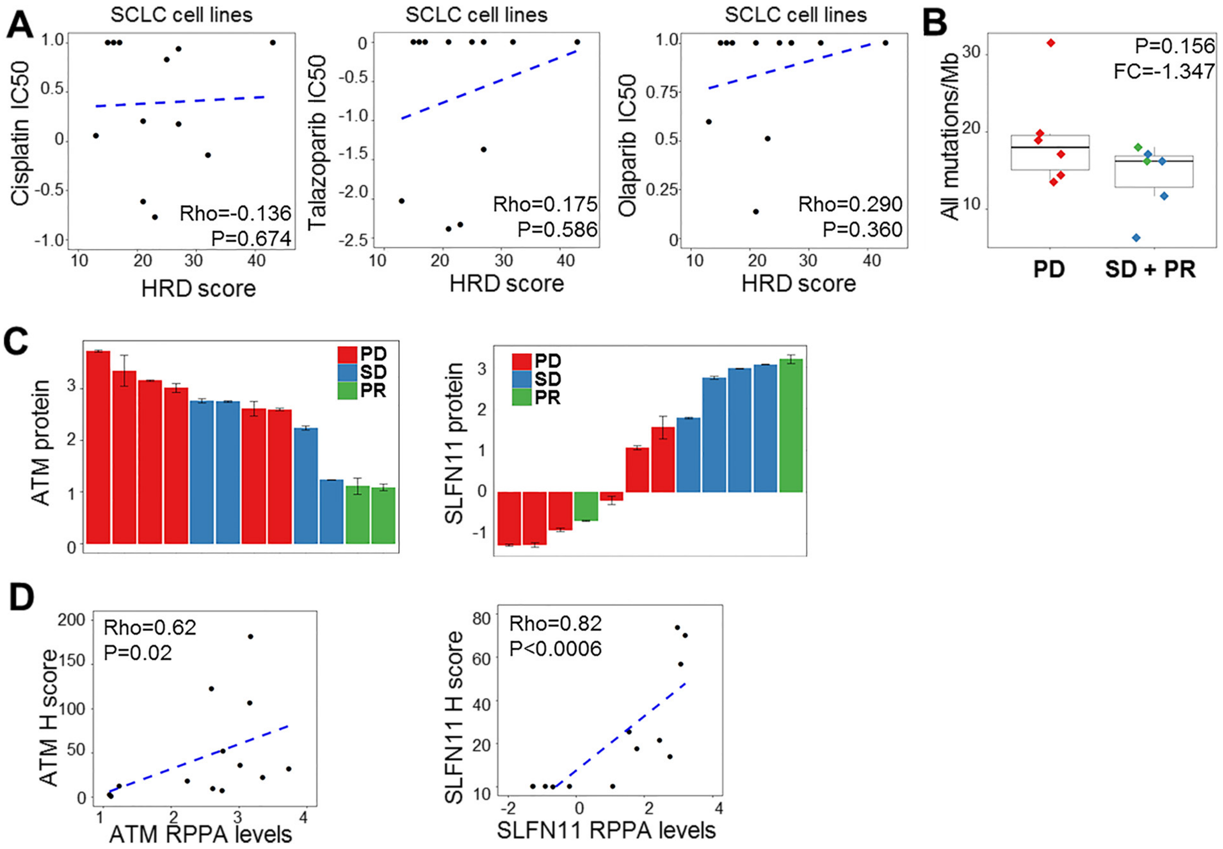

Supplementary Figure 2: HRD Score and Mutation Burden are not Associated with PARP Inhibitor Response in SCLC Cell Lines or Tumors. **A.** Myriad HRD score is not correlated with IC_{50} values for cisplatin, talazoparib, or olaparib in 12 SCLC cell lines. **B.** The FoundationOne mutation burden, per megabase (MB) and PDX response, does not correlate with response. **C.** ATM and SLFN11 protein expression levels in the PDX samples color-coded by response. **D.** ATM and SLFN11 protein levels are correlated with H scores.

A

Gene Symbol	Talazoparib IC50	Cisplatin IC50
SLFN5	0.08	-0.25
SLFN11	**-0.49***	**-0.60***
SLFN12	0.06	0.05
SLFN13	0.15	0.11
SLFN14	-0.22	0.02

*P<0.005

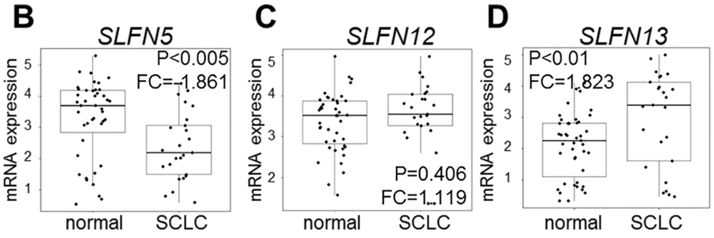

Supplementary Figure 3: SLFN Family Member Expression and Sensitivity in SCLC. **A.** SLFN family member mRNA expression is correlated with both talazoparib and cisplatin IC_{50} values in SCLC cell lines. **B–D.** Expression of *SLFN 5, SLFN12,* and *SLFN13* mRNA in normal tissues compared to SCLC tumors.

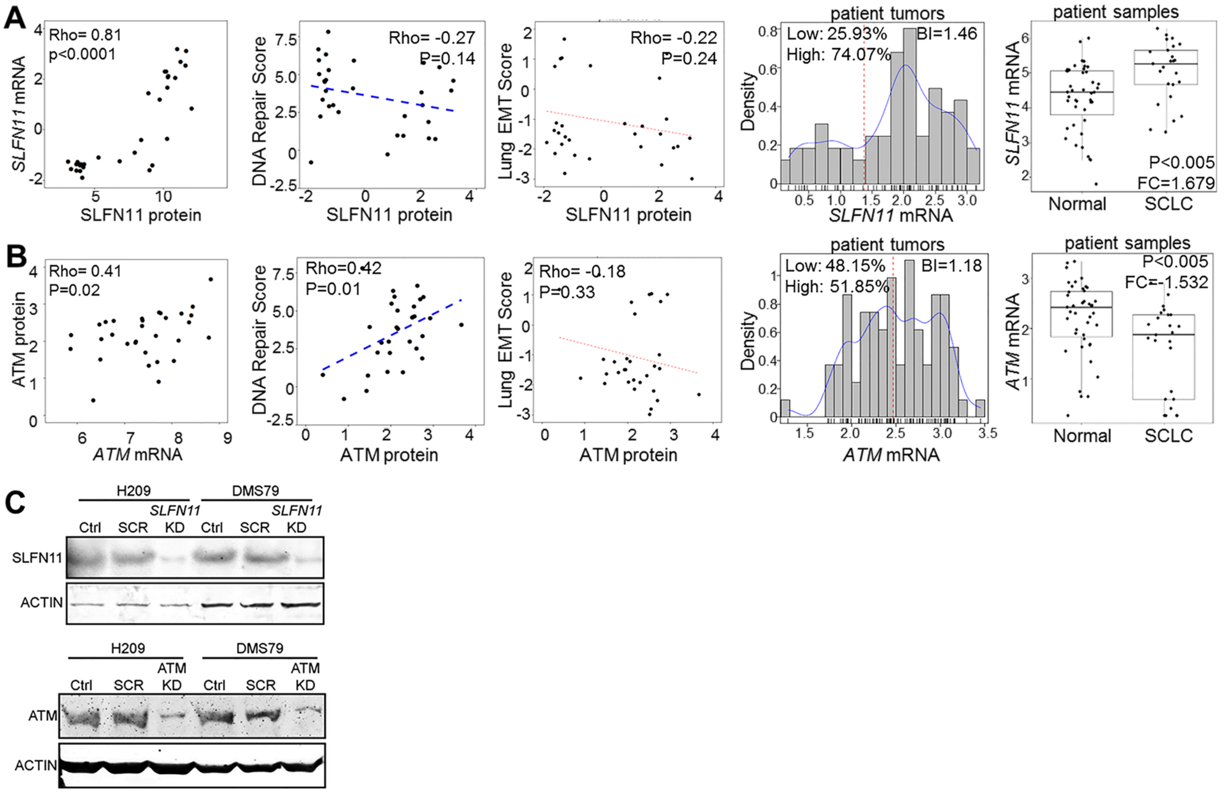

Supplementary Figure 4: Relationship between SLFN11 and ATM Protein Levels and DNA Repair Score or EMT Score in SCLC Cell Lines. **A** and **B.** SLFN11 (A) and ATM (B) mRNA and protein expression levels are concordant in SCLC cell lines. SLFN11 expression is not correlated with DNA repair score or EMT score in cell lines (A). ATM expression is correlated with DNA repair score but not EMT score. Both SLFN11 (A) and ATM (B) are bimodal in treatment-naïve SCLC patient tumors. SCLC patient tumors have elevated *SLFN11* (A) and reduced *ATM* (B) mRNA levels compared with adjacent normal tissues. **C.** Silencing of SLFN11 and ATM with siRNA effectively reduces protein expression (C).

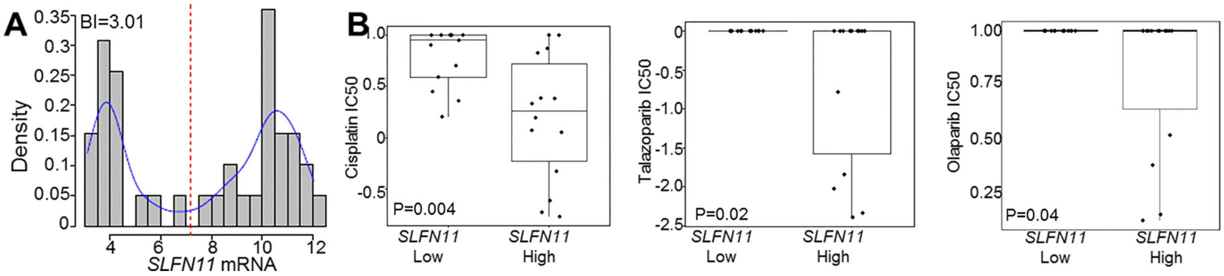

Supplementary Figure 5: *SLFN11* mRNA Levels Are Correlated with Drug Sensitivity in SCLC. A. Similar to SFLN11 protein, *SLFN11* mRNA is bimodally distributed in SCLC cell lines. **B.** Cell lines with higher *SLFN11* mRNA expression levels demonstrate greater sensitivity to cisplatin, talazoparib, and olaparib.

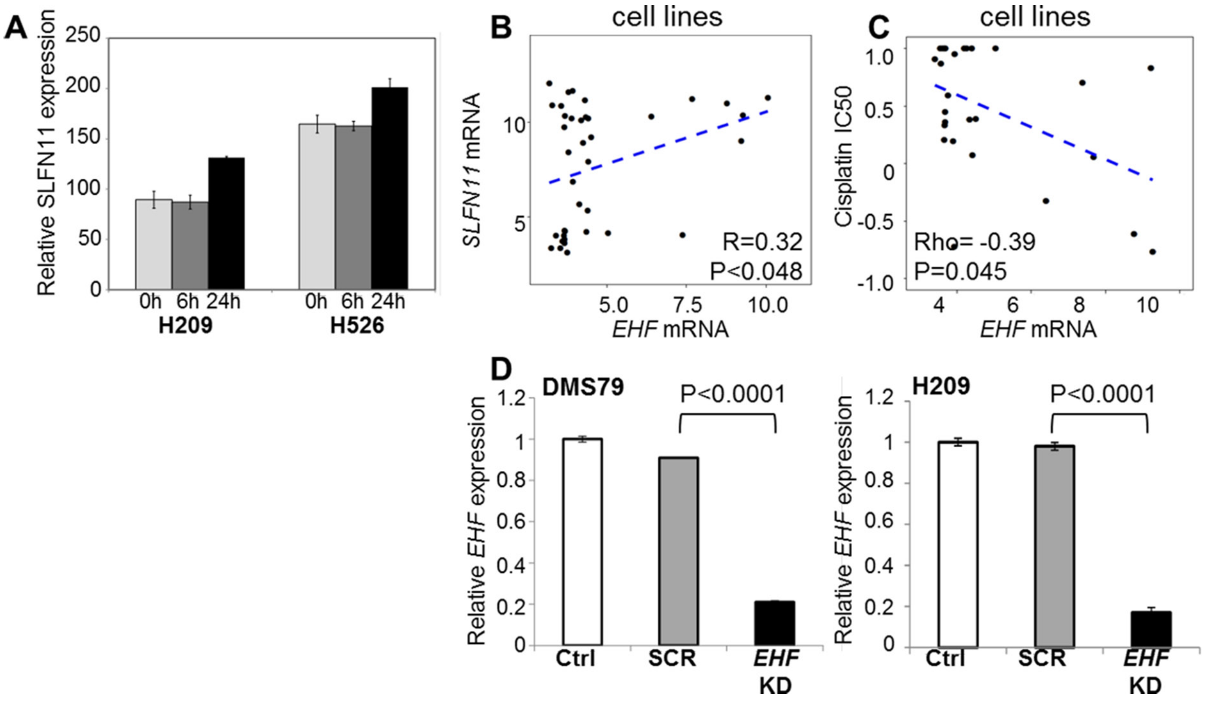

Supplementary Figure 6: *EHF Is* Correlated with *SLFN11* Levels and Cisplatin Sensitivity in SCLC. A. H209 and H526 SCLC cell lines were treated with Interferon beta-1A (1000 IU) for 0, 6h or 24h and SLFN11 protein levels were measured. **B.** *EHF* is correlated with *SLFN11* expression in cell lines. **C.** *EHF* levels are associated with sensitivity to cisplatin. **D.** Validation that silencing of *EHF* effectively reduces *EHF* expression.

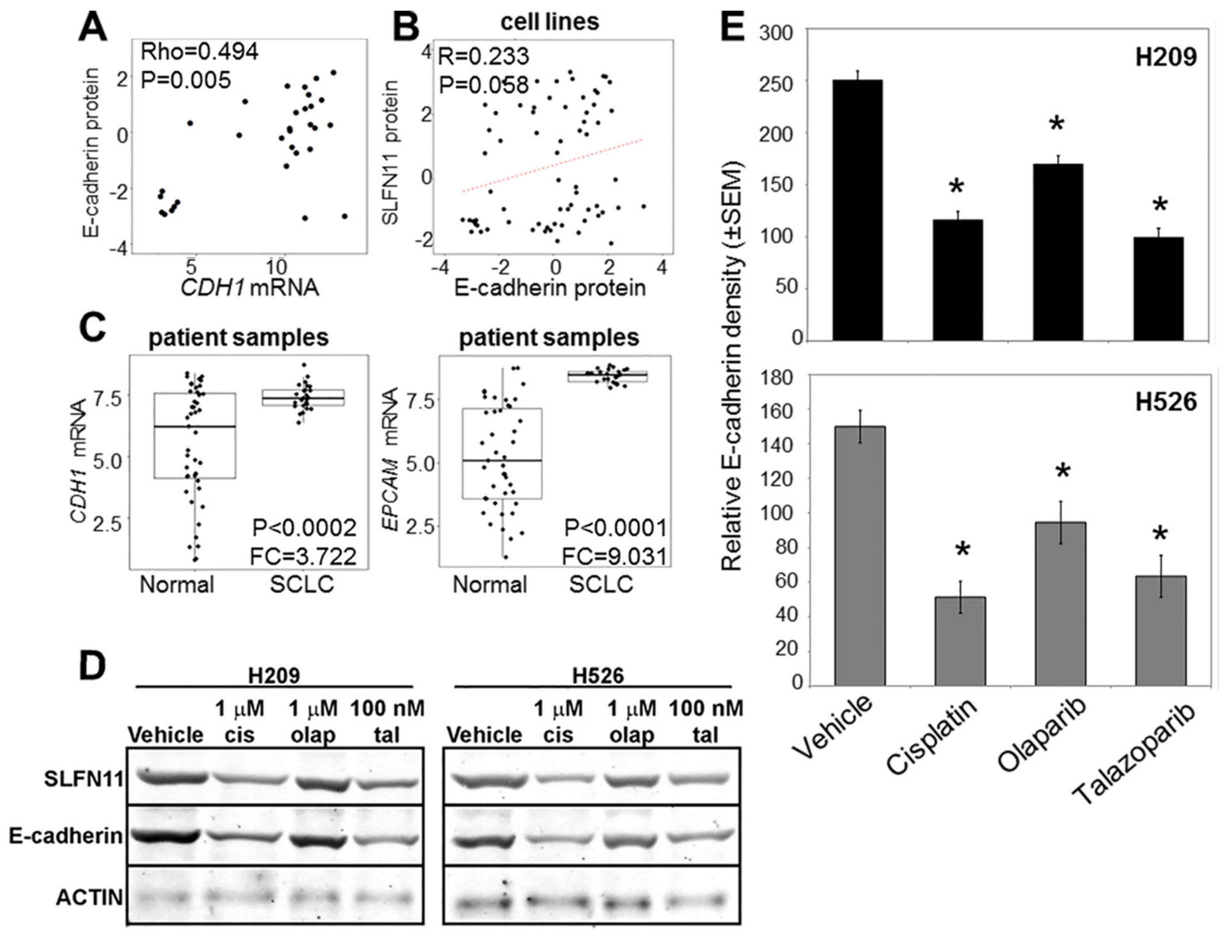

Supplementary Figure 7: E-cadherin is Elevated in SCLC Tumors and Associated with SLFN11 in Cell Lines. **A.** E-cadherin protein and *CDH1* mRNA expression levels are concordant in 51 SCLC cell lines. **B.** E-cadherin and SLFN11 protein expression levels are correlated in SCLC cell lines. **C.** *CDH1* and *EPCAM* mRNA expression levels are higher in SCLC patient tumors than in adjacent normal tissues. **D.** Representative western blots of H209 and H526 cell lines treated with 1 µM cisplatin, 1µM olaparib, or 100 nM talazoparib for 72h. **E.** Treatment of H209 and H526 SCLC cell lines with 1 µM cisplatin, 1µM olaparib, or 100 nM talazoparib reduced E-cadherin levels compared with vehicle-treated cells. *, P<0.0001.

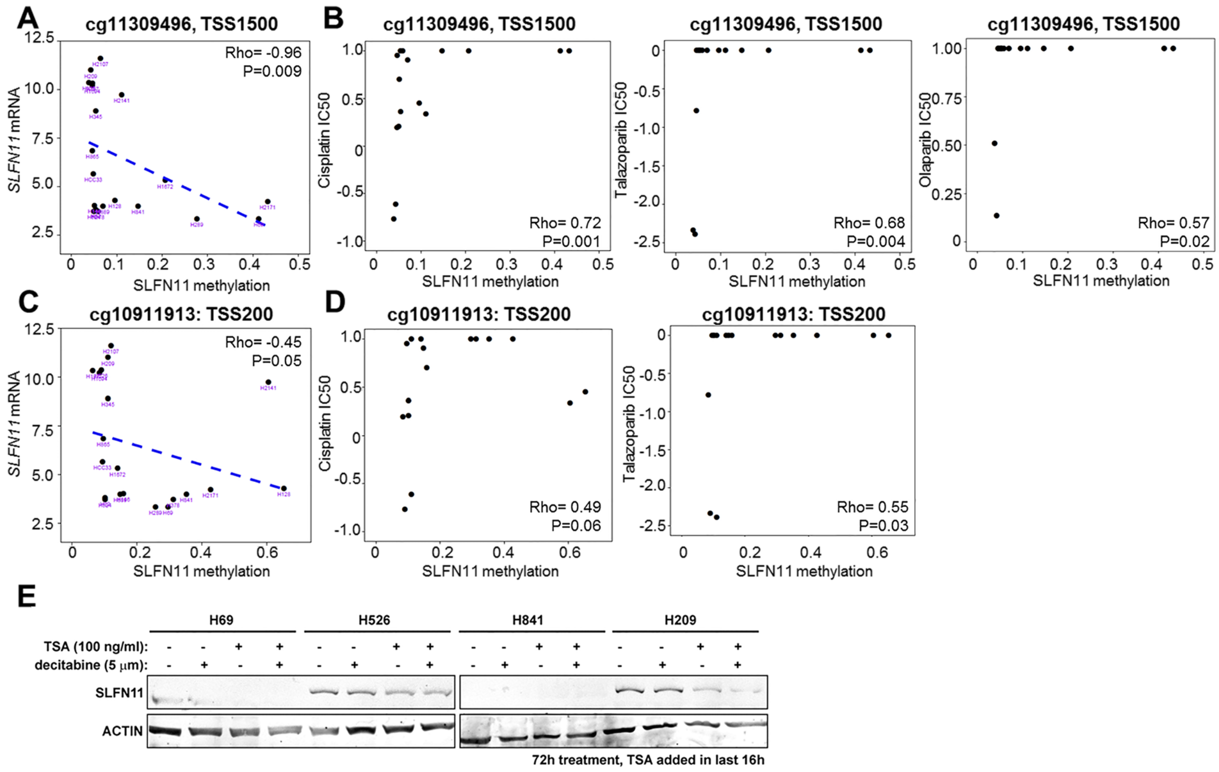

Supplementary Figure 8: SLFN11 Promoter Methylation Is Associated with Cisplatin and PARP Inhibitor Resistance in SCLC Cell Lines, Demethylation Is Insufficient to Upregulate SLFN11. A and **C.** SLFN11 methylation sites are correlated with mRNA expression in 35 SCLC cell lines. **B** and **D.** These same methylation sites are correlated with resistance to cisplatin, talazoparib, and olaparib. **E.** Demethylation with 5 µM decitabine and/or inhibition of histone deacetylase activity with 100 ng/ml trichostatin A (TSA) does not increase SLFN11 levels.

Supplementary Table 1: Drug sensitivity of knockdown cell lines

Drug	Cell line	IC50 (µM)
Cisplatin	DMS79	0.352
Cisplatin	DMS79SCR	0.440
Cisplatin	DMS79ATMKD	0.063
Cisplatin	DMS79SLFNKD	9.289
Cisplatin	H209	1.387
Cisplatin	H209SCR	1.650
Cisplatin	H209ATMKD	0.411
Cisplatin	H209SLFNKD	9.600
Talazoparib	DMS79	0.010
Talazoparib	DMS79SCR	0.014
Talazoparib	DMS79ATMKD	0.004
Talazoparib	DMS79SLFNKD	>1.000
Talazoparib	H209	0.018
Talazoparib	H209SCR	0.033
Talazoparib	H209ATMKD	0.001
Talazoparib	H209SLFNKD	>1.000
Olaparib	DMS79	0.656
Olaparib	DMS79SCR	0.623
Olaparib	DMS79ATMKD	0.072
Olaparib	DMS79SLFNKD	9.600
Olaparib	H209	1.620
Olaparib	H209SCR	2.820
Olaparib	H209ATMKD	0.071
Olaparib	H209SLFNKD	9.600

Supplementary Table 2: Correlation analysis of ETS family members with SLFN11 and PARP1 mRNA expression and with cisplatin, talazoparib, and olaparib IC50 values (*P<0.05, **P<0.01, ***P<0.005)

Gene Symbol	*SLFN11 (Rho)*	*PARP1 (Rho)*	cisplatin IC50 (Rho)	talazoparib IC50 (Rho)	olaparib IC50 (Rho)
ETV2	0.364*	-0.15	0.048	0.006	0.109
EHF	0.318*	-0.284	-0.389*	-0.352	-0.367
ELF1	0.294	-0.282	-0.331	-0.430*	-0.534***
ETS2	0.265	-0.164	-0.084	0.122	0.066
ELF3	0.213	0.024	-0.213	-0.345	-0.428*
ELK1	0.209	0.005	-0.126	0.026	0.071
SPIB	0.194	-0.097	-0.460*	-0.528*	-0.475**
SPIC	0.181	-0.456***	0.389*	0.235	0.267
ETV6	0.177	-0.093	-0.106	0.017	0.019
ETV3	0.164	-0.307	0.023	0.153	0.312
ELF2	0.154	0.249	-0.024	-0.105	-0.093
ETV5	0.137	0.033	-0.008	0.247	0.371
SPI1	0.08	-0.286	0.071	0.055	0.136
ELF5	0.077	-0.285	0.046	-0.227	-0.325
ETV7	0.075	-0.102	-0.406*	0.393*	-0.354
ERG	0.06	-0.102	-0.312	-0.355*	-0.505**
GABPA	0.032	0.495***	-0.171	0.023	0.146
ERF	0.025	-0.413**	0.107	0.056	0.150
FEV	0.017	-0.015	0.062	-0.015	0.128
ETV4	-0.009	-0.451***	-0.023	0.205	0.261
ETS1	-0.047	-0.325**	0.186	0.097	0.038
SPDEF	-0.078	-0.276	0.257	0.255	0.194
ELK3	-0.114	-0.178	-0.239	0.061	0.072
ETV1	-0.197	0.272	0.143	0.087	0.075
ELF4	-0.201	-0.146	0.272	0.16	0.181
ELK4	-0.259	0.310*	0.023	0.356	0.261

ETS genes are ranked by decreasing Spearman correlation coefficient in the SLFN11 column.

Supplementary Table 3: Expression of ETS genes in SCLC patient tumors compared to normal, adjacent tissues

Elevated in SCLC tumors		Reduced in SCLC tumors	
**Gene Symbol**	**P-value**	**Gene Symbol**	**P-value**
ELF2	P<0.0001	*ELK3*	P<0.0001
ELF3	P<0.0001	*ETS1*	P<0.0001
EHF	P<0.002	*ELF1*	P<0.0004
ETV7	P<0.005	*SPI1*	P<0.005
		ETV3	P<0.007
		SPDEF	P=0.020
		ERG	P=0.022
		ETV6	P=0.042
		ERF	P=0.044

ETS transcription factors are ranked by decreasing P-values.
